# 3-T MRI and clinical validation of ultrasound-guided transperineal laser ablation of benign prostatic hyperplasia

**DOI:** 10.1186/s41747-021-00239-9

**Published:** 2021-09-17

**Authors:** Guglielmo Manenti, Tommaso Perretta, Antonello Calcagni, Donatella Ferrari, Colleen P. Ryan, Federico Fraioli, Rosaria Meucci, Andrea Malizia, Valerio Iacovelli, Enrico Finazzi Agrò, Roberto Floris

**Affiliations:** 1 Department of Biomedicine and Prevention, University of Rome “Tor Vergata”, 00133 Rome, Italy; 2 Department of Diagnostic Imaging and Interventional Radiology, University of Rome “Tor Vergata”, Viale Oxford 81, 00133 Rome, Italy; 3 Department of Neuromotor Physiology, Fondazione Santa Lucia IRCCS, Rome, Italy; 4 Department of Urology, University of Rome “Tor Vergata”, 00133 Rome, Italy

**Keywords:** Laser therapy, Lower urinary tract symptoms, Magnetic resonance imaging, Prostatic hyperplasia

## Abstract

**Background:**

Transperineal laser ablation (TPLA) of the prostate is a novel, mini-invasive option for men with lower urinary tract symptoms (LUTS) due to benign prostate hyperplasia (BPH). Our aim was to assess the impact of ultrasound-guided TPLA regarding urodynamic improvement and sexual function, monitoring clinical data, postprocedural complications and imaging findings at 3-T multiparametric magnetic resonance imaging.

**Methods:**

Forty-four patients aged ≥ 50 affected with moderate to severe LUTS (International Prostate Symptoms score ≥ 12) due to benign prostatic obstruction and refractoriness, intolerance or poor compliance to medical therapies underwent US-guided TPLA between May 2018 and February 2020. Clinical measurements included PSA, uroflowmetry, sexual function assessment (using the International Index of Erectile Function and Male Sexual Health Questionnaire-Ejaculatory Dysfunction short form) and quality of life questionnaire. Adverse events were evaluated using the Clavien-Dindo scale. Volume changes were measured by MRI and automatic segmentation software during 1-year follow-up. Registration: NCT04044573 – May 5th, 2018, https://www.clinicaltrials.gov

**Results:**

MRI assessed the changes over time with a 53% mean reduction of adenoma volume and 71% of the ablated area, associated with clinical and functional improvement and resolution of LUTS in all cases. Five of 44 patients (11.3%) had urinary blockage due to clots and required re-catheterisation for 2 weeks. The overall adverse event rate was 7%.

**Conclusion:**

US-guided TPLA performed as a safe, manageable and effective treatment for LUTS. It could be considered an alternative effective mini-invasive procedure to standard treatments for BPH in the outpatient setting.

## Key points

- Ultrasound-guided transperineal laser ablation (TPLA) represents an option for the treatment of benign prostate hyperplasia.
- Ultrasound-guided TPLA preserves urological and sexual function.
- US-guided TPLA provides a treatment option for patients otherwise ineligible for standard treatments.

## Background

Benign prostate enlargement is a common disease amongst elderly men. It may cause functional obstruction of the bladder outlet (BPO), which is the major cause of lower urinary tract symptoms (LUTS) [1, 2]. Men who have moderate-to-severe LUTS can benefit from pharmacological treatment as single or combination therapy [3].

However, these are not well tolerated due to side effects, such as decreased libido, impotence and decreased ejaculate [2]. If drugs are insufficiently effective or not tolerated, minimally invasive approaches can be performed [4]. Current mini-invasive methods include transurethral resection of the prostate (TURP), Holmium Laser Enucleation of the Prostate (HoLEP) and photovaporisation of the prostate. Amongst these, TURP is still considered the cornerstone of LUTS/BPO minimally invasive treatment in men with a prostate size of 30–80 mL [5]; however, it is associated with the prevalence of important complications such as retrograde ejaculation, in more than 30% of cases [6–8]. In the same regard, HoLEP causes retrograde ejaculation in 70% of cases, whereas this complication has been reported to be 63% for photovaporisation of the prostate using Nd:YAG laser [9]. These procedures have been proposed as an alternative to TURP in order to reduce invasiveness and hospitalisation time.

In selected cases, simple prostatectomy techniques should be considered such as open, laparoscopic or robotic-assisted prostatectomy. These procedures generally require regional (*i.e*., spinal, epidural) or general anaesthesia and varying duration of hospital stay. They require adequate selection of patients who may be subjected to considerable surgical and perioperative morbidity [4]. This makes standard of care treatments unavailable in health emergencies such as during the recent novel coronavirus 2019, COVID-19, outbreak.

Recently, a novel option has been described for BPO: transperineal interstitial laser ablation (TPLA) of the prostate [10, 11], which consists of thermal ablation of the prostate adenoma, reducing the tissue volume in the transitional zone of the prostate. This method creates immediate tissue damage related to high local temperature and delayed extensive coagulative necrosis generally leading to final post-necrotic reabsorption of the treated volume.

This study aims to evaluate ultrasound (US9-guided TPLA in patients affected by LUTS. Evaluation included the perioperative and short-term outcomes up to 12 months, including the assessment of postprocedural complications as well as urinary and sexual function. Prostate volume reduction and coagulative necrosis extension over time was calculated using MRI data processing by an automated segmentation software.

## Methods

### Study group

Male patients prospectively included in this study were aged between 59 and 82 years (72 ± 6.6, mean ± standard deviation) from May 2018 to February 2020. The Institutional Review Board approved this prospective study, procedures were conducted with ethical standards, in accordance with the guidelines of the Declaration of Helsinki, and written informed consent was obtained from all enrolled subjects. Men who over 50 years had moderate to severe LUTS due to BPO with an International Prostate Symptom Score, (IPSS) score ≥ 12, prostate volume ≥ 30 mL and ineffectiveness of combined medical therapies because of lack of efficacy, intolerance, or poor compliance were enrolled in the study. All patients were previously treated with alpha-adrenergic receptor blockers and 5-alpha-reductase inhibitors. Patients expressed dissatisfaction with the effectiveness of the treatment. Men with a history of urethral stricture or prostatic surgery, neurogenic bladder, previous prostatic or bladder cancer, with multiparametric magnetic resonance imaging (MRI) signs of prostate malignancy confirmed by biopsy investigation, with indwelling catheter or with prostatic median lobe larger than 10 mm were excluded from the study. Inclusion and exclusion criteria are reported in Table 1.

**Table 1:** Inclusion and exclusion criteria

Inclusion criteria	Exclusion criteria
1. Age ≥ 50 years 2. International Prostate Symptoms Score ≥ 12 3. Prostate volume ≥ 30 mL 4. Intolerance or/and lack of results of combined medical treatment (alpha-adrenergic receptor blockers and 5-alpha-reductase inhibitors)	1. History of urethral stricture or prostatic surgery 2. Signs of malignancy at magnetic resonance imaging, confirmed by biopsy investigation 3. Neurological disorders (neurogenic bladder) 4. Large median lobe (> 10 mm on longitudinal axis) 5. Indwelling catheter 6. Previous diagnosis of bladder or prostate cancer

All patients were uncomfortable with more invasive treatments at the time of enrolment from TPLA treatment. Patients underwent a preoperative multiparametric contrast-enhanced MRI (gadobutrol 1.0 mmol/mL, 0.1 ml/kg; Bayer Healthcare, Berlin, Germany), according to the sequence protocol shown in Table 2. MRI was evaluated by a radiologist with more than 15 years of experience in urogenital radiology to exclude the presence of suspicious lesions according to the *Prostate Imaging Reporting and Data System* version 2.1 [12] and to assess the morphological characteristics of benign prostate enlargement and prostate volume.

**Table 2:** 3-T multiparametric magnetic resonance imaging protocol for patient selection

Sequences	Plane	Time (min:s)	Repetition time (ms)	Echo time (ms)	Flip angle (°)	Number of slices	Slice thickness (mm)	Pixel size (mm)
1. T2-weighted turbo spin-echo	Sag	2:45	3,293	110	90	25	3	0.7 × 0.9
2. T2-weighted turbo spin-echo	Ax	5:42	3,000	110	90	24	3	0.6 × 0.9
3. Diffusion-weighted imaging^a^	Ax	6:24	3,459	88	90	24	3	2.4 × 2.8
4. T2-weighted turbo spin-echo	Cor	5:06	3,000	110	90	24	3	0.5 × 0.8
5. T1-weighted Dixon dynamic (30 phases)	Ax	3:35	3.7	1.37	10	25	3	1.5 × 1.5
6. T1-weighted turbo spin-echo	Ax	2:21	561	8	90	31	5	1.0 × 1.0

^a^*b*-values: 0, 1,000, 1,500, 2,000 ms. *Ax* Axial, *Cor* Coronal, *Sag* Sagittal. Patient follow-up was performed using only the sequences here numbered from 1 to 4

Before the procedure, all the patients underwent blood testing including coagulation measurements. They were requested to complete the following questionnaires: IPSS, International Index of Erectile Function (IIEF5) [13], Male Sexual Health Questionnaire - Ejaculatory Dysfunction (MSHQ-EjD) short form [14], and International Quality of Life (I-QoL) [15]. PSA and uroflowmetry parameters (*Q*_max_ and postvoid residual, PVR) were registered preoperatively. Complications were reported with the Clavien-Dindo rating [16]. A single-item visual analogue scale (VAS) pain score [17] and McGill Pain Questionnaire [18] were obtained for all the patients at the end of the treatment. Pain was reported as “external”, “steady”, “discomforting” and “dull” according to the McGill Pain Questionnaire in 87% of treated patients and lasting 48–96 h after the procedure. Major complications and minor complications were reported and classified as intraoperative, perioperative (within 24 h) and postoperative (within 30 days). No intra-operatory or major complications were observed. In 5/44 patients (11.3%), a catheter was repositioned after removal for a urinary blockage and urinary clots at day 7 urologic assessment and maintained for 7 days further. These cases were considered minor postoperative complications. Major complications and minor complications were reported and classified as intraoperative, perioperative (within 24 h), postoperative (within 30 days). No intra-operatory or major complications were observed. In 5/44 patients (11.3%), a catheter was repositioned after removal for a urinary blockage and urinary clots at day 7 urologic assessment and maintained for 7 days further. These cases were considered minor postoperative complications.

### TPLA procedure

A radiologist with more than 10 years of experience in biopsy procedures performed SoracteLite^TM^ TPLA treatment with transrectal US guidance. The US machine (MyLab9 eXP Platform, Esaote S.p.A. Genoa, Italy) was connected to a support planning system (Echolaser Smart Interface, Elesta S.p.A., Calenzano, Florence, Italy) and used to guide the procedures through a transrectal ultrasound probe. Patients were placed supine in the lithotomy position. The bladder and urethra were continuously irrigated by saline through a three-way catheter to avoid any thermal damage. Perineal and periprostatic local anaesthesia was administered (20 mL lidocaine solution 2%); none of the patients underwent moderate conscious sedation.

Chiba needles 21 G (Elesta S.p.A., Calenzano, Florence, Italy) were inserted, with a dedicated guidance device for a biplane linear probe into the perineal space inside the prostatic gland and then they were used as introducers. Up to two needles for each lobe were positioned depending on the shape and volume of the prostate (one fibre per lobe if the prostate volume was < 45 mL). If multiple in one lobe, these were consequently positioned at a distance of 8–10 mm from each other inside the central gland adenoma. Needles were positioned as parallel as possible to the prostatic longitudinal axis. Needles tip placement on the axial plane was tailored according to the relation of the urethral position and its longitudinal width to generate a symmetric cavity of ablation, to reduce urethral stromal compression and to shift the urethral lumen as close as possible to the midline. Positions were confirmed in real-time and eventually modified using the biplane US device. In the lumen of the needles bare optic quartz fibres were then introduced with a diameter of 272 μm (Oberon GmBH, Wildau, Germany), that protruded from the applicator tip by 10 mm and were connected to a multisource laser machine operating at 1064 nm (EchoLaser X4, ElEn S.p.A. Calenzano, Florence, Italy). Distances over 10 mm from the urethral wall, 15 mm from the bladder and 10 mm from the prostatic capsule outer edge were maintained.

**Fig. 1:**
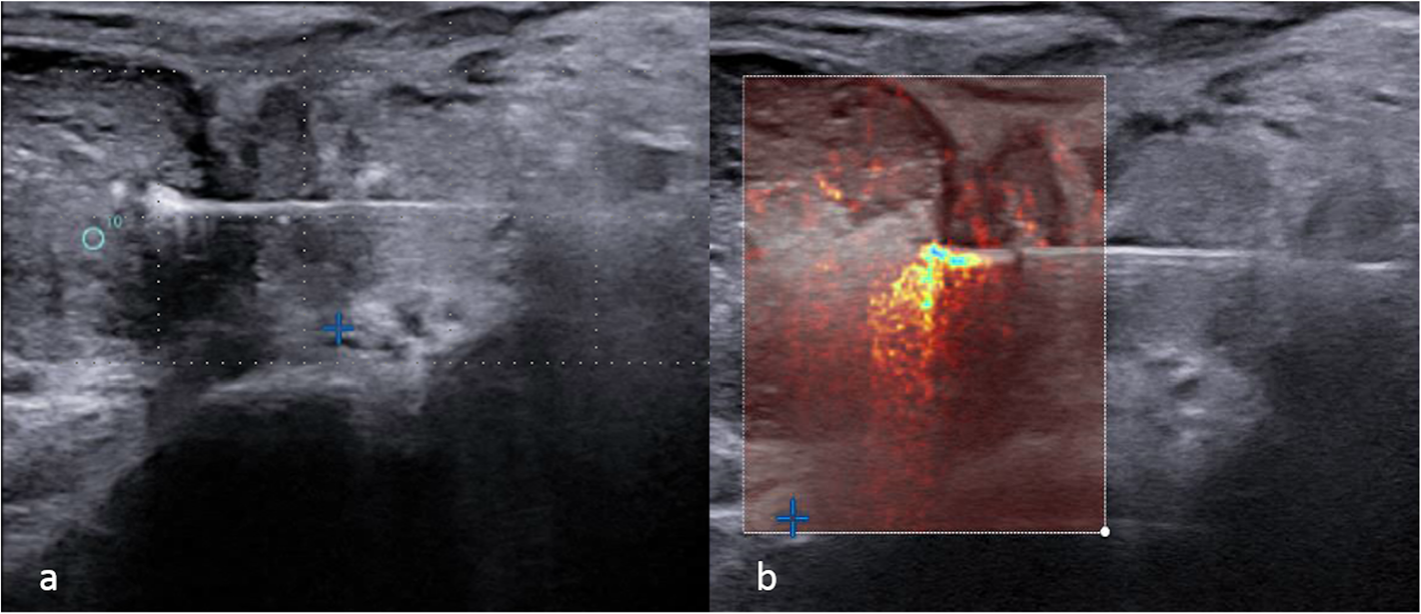
**a** Transrectal ultrasound imaging of transperineal laser ablation with gas bubble formation during the procedure. **b** Power Doppler imaging with Doppler effect related to water molecule movement into the ablated tissue

A fixed power of 3 W was applied during the procedure after an initial 2-min 5-W pulse ablation was adapted to prostatic volume (not more than 2 ablation sessions per each treatment). One pullback manoeuvre (retraction of the fibres by 1 cm in the longitudinal axis) was performed to widen the ablative area. Procedural ablation time ranged from 400 to 600 seconds for each fibre. Each fibre operated at 1,800 J, with a maximum total energy delivery of 7,200 J. Laser light comes out of the fibre tip and turns into thermal energy capable of destroying tissue. The temperature near the tip of the fibre reaches 100 °C, causing vaporisation (Fig. 1) in the target tissue area and cell death through protein denaturation and coagulative necrosis [19]. The shape of the induced necrosis area is elliptical, with a long axis of 16–18 mm and a short axis of 10–12 mm for each laser fibre. During the procedure, energy delivery parameters are monitored by the operator through a display on the laser machine and the progress of ablation was monitored by US. Thermocouples were not used. The mean operative time was calculated from the administration of local anaesthesia to the end of ablation, considered ultimate when are reached the 7,200 J or when gas fills the target area.

Antibiotic prophylaxis (levofloxacin 500 mg) was administered 1 h before and for 5 days after the treatment. In standard cases, neither any thromboprophylaxis nor single antiplatelet therapy suspension was required. An intraprocedural single dose of dexamethasone (8 mg) was administered intravenously to reduce postprocedural prostatic oedema. Pain was effectively managed by use of acetaminophen 1,000 mg.

Prednisone 25 mg was administered for 5 days with subsequent dose tapering, to hold off oedema related to thermal effect. Alpha blockers were continued for 30 days after TPLA.

One hour after the procedure, patients underwent 3-T biparametric MRI (using only the first four sequences listed in Table 2) to evaluate the extension of the coagulation zone at each laser fibre tip. After a couple of hours, the patients were discharged with a bladder catheter that was removed after 1 week. In case of post-catheter removal retention, a new 16-F Foley catheter was placed and kept for 7 more days.

### Follow-up

MRI follow-up was planned after 1 month, and both clinical and imaging follow-up after 3 months, 6 months, and 12 months from the treatment. The outcomes analysed at 3, 6, and 12 months included urodynamic parameters (*Q*_max_ and PVR), sexual function (IIEF5), ejaculation function (MSHQ-EjD), symptoms and quality of life evaluation (IPSS and I-QoL). At 1, 3, 6, and 12 months, each patient also underwent biparametric MRI, using only the first four sequences listed in Table 2. To monitor the necrotic area volume and to assess the prostatic volume decrease and the ablated cavum size using the Philips IntelliSpace Portal 7.0 Multi-Modality Tumor Tracking software, by the same radiologist who performed the pre-treatment analysis.

The main radiological findings investigated and annotated during MRI follow-up of TPLA were visualisation of needle track, presence of periurethral oedema, assessment of posttreatment cavity, urethra morphology preservation and reduction of prostate volume. The 12-month clinical outcomes and volume quantification analysis (whole prostate and extension of laser ablation) were reported as indicators of procedural efficacy.

Morbidity was assessed using the Clavien-Dindo system [16], as indicators of procedural safety.

### Statistical analysis

Analyses were conducted using two-tailed testing. Continuous variables were analysed using the Mann–Whitney *U* test whilst categorical variables were compared using the *χ*^2^ test; *p*-values were considered statistically significant when being lower than 0.05. All calculations were performed using Stata 15 (StataCorp 2017, College Station, TX, USA) and R 3.6.1 (R Foundation for Statistical Computing, Vienna, Austria) software.

## Results

All 44 consecutive patients (age 72.1 ± 6.6, mean ± standard deviation) were treated according to the protocol and completed the 12-month follow-up. Baseline and 12-month patient characteristics are reported in Table 3.

**Table 3:** Clinical and subjective patient data (preprocedural and at 12-month follow-up)

Parameters	Preprocedural	12-month follow-up
Prostate specific antigen (ng/mL)	7.3 ± 1.8	2.1 ± 0.8
Prostate volume (mL)	102.42 ± 36.3	48.12 ± 19.2
Flow rate estimation, (mL/s)	7.6 ± 4.2	16.2 ± 4.9
Postvoiding urine residual volume (mL)	138.4 ± 40.8	18.8 ± 8.5
International index of erectile function 5	21 ± 4	22 ± 3
Male Sexual Health Questionnaire for Ejaculatory Dysfunction (MSHQ-EjD)	4.9 ± 3.7	7.7 ± 3.2
International prostate symptom score	18.5 ± 5.5	6.2 ± 3.8
Quality of life	5.8 ± 1.4	2.1 ± 1.1
Clavien-Dindo scale	0	0

### Baseline

Prostate volume was 102.4 ± 36.3 mL (mean ± standard deviation), ranging from 36 to 160 mL. All the patients suffered from moderate to severe LUTS with an IPSS of 18.5 ± 5.5 (mean ± standard deviation) and an I-QoL score of 5.8 ± 1.4. Sexual function was self-evaluated using the IIEF-5 score 21.2 ± 4.0 (mean ± standard deviation), reflecting a relatively normal erection. On the contrary, before the procedure, 30 patients (68%) reported altered ejaculation at MSHQ-EjD. Preoperative uroflowmetry showed a mean *Q*_max_ of 7.6 ± 4.2 mL/s and a PVR of 138.4 ± 40.8 mL (mean ± standard deviation).

In 37 patients with symmetric prostate and prostate volume between 90 and 160 mL, 4 laser fibres were used. In 4 patients with asymmetric lobes (*e.g.*, the dominant lobe > 20% in volume compared to the contralateral lobe) and prostate volumes from 60 to 90 mL, 3 fibres were used. In 3 patients with prostatic lobe symmetry and prostate volumes from 30 and 60 mL, 2 fibres were used.

The mean operative time was 28.2 min ± 10.6 (range 21–75 min).

### Follow-up

**Fig. 2:**
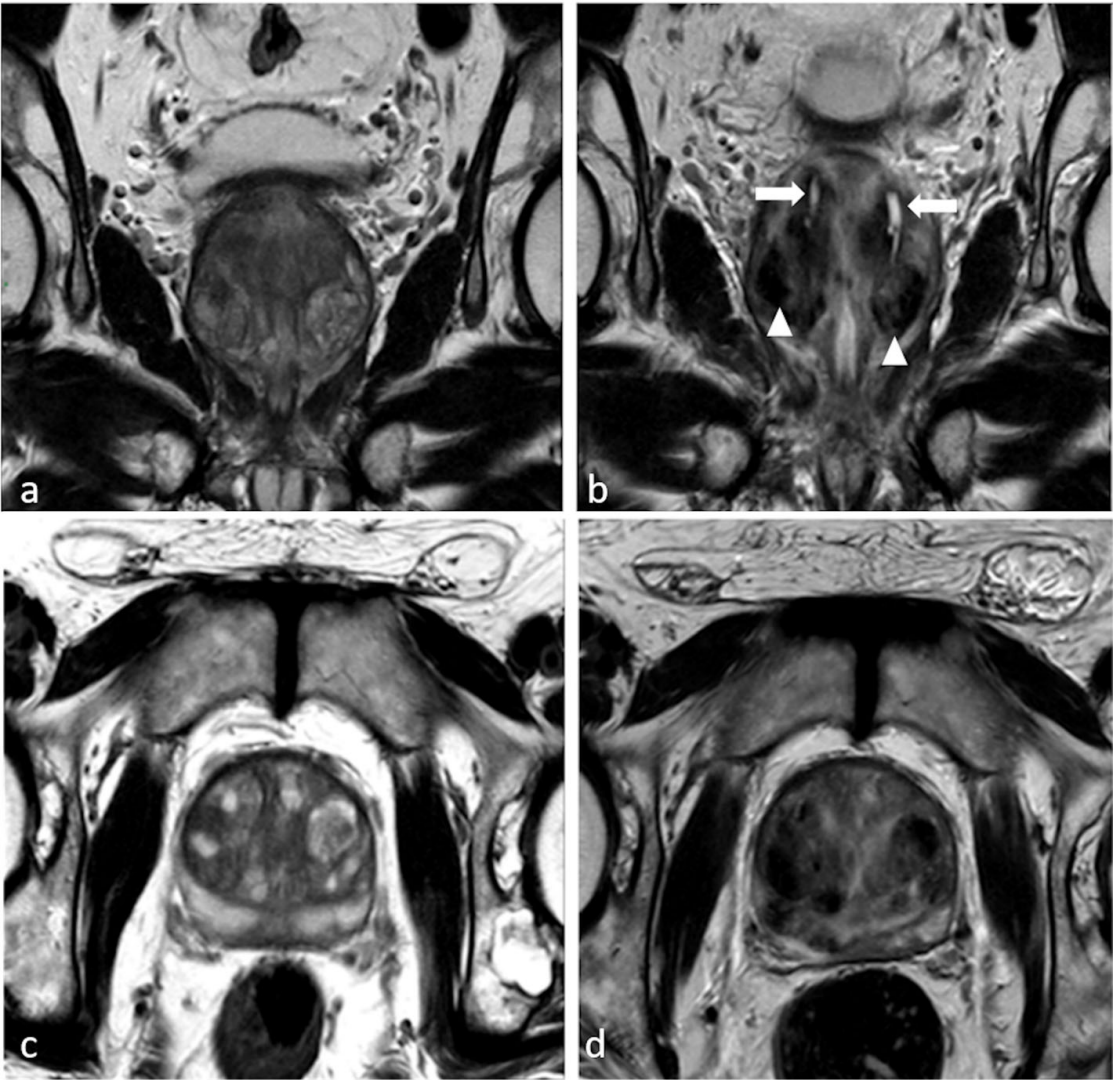
Large bilateral symmetrical bilobar adenoma in a 62-year-old patient before treatment, on magnetic resonance imaging T2-weighted sequences along the coronal (**a**) and axial (**c**) planes. The same sequences, along the coronal (**b**) and axial (**d**) planes, performed immediately after transperineal prostate laser ablation show only longitudinal linear hyperintensities (*arrows*) surrounded by hypointense elliptical-shaped charred tissue (*arrowheads*) at the site of laser fibre tracks. At this time, no signs of coagulative necrosis are yet visible

**Fig. 3:**
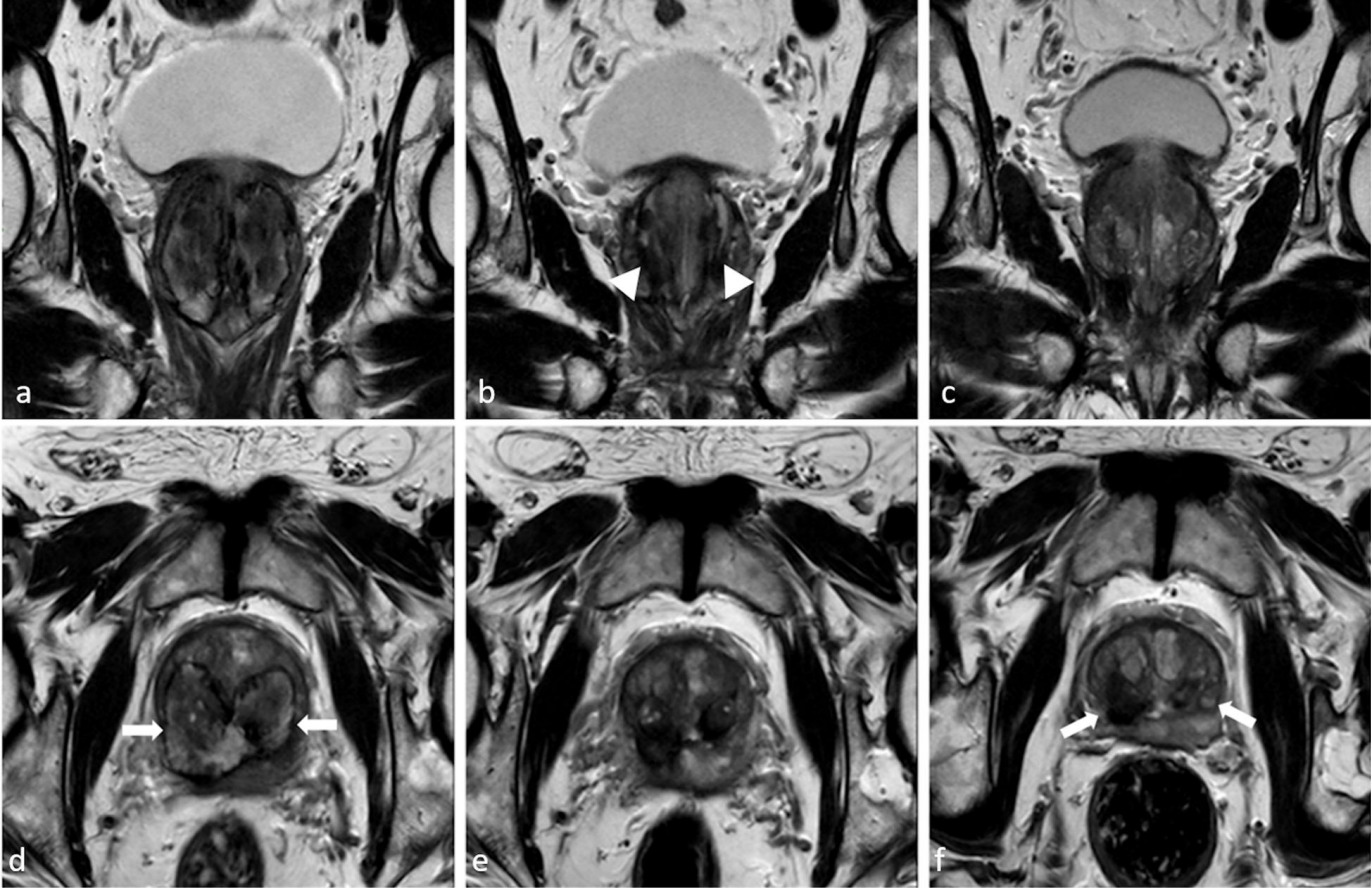
Follow-up from month 3 to month 12 in the same patient shown in Figs. 1 and 2: T2-weighted sequences along coronal and axial planes. **a**, **d** Month 3: large bilateral and symmetrical necrotic cavitations (*arrows*); prostate volume 72 cc. **b**, **e** Month 6: Fluid-filled cavities are reabsorbed. Tracks from fibre applicators are slightly visible (*arrowhead*). Urethral morphology is preserved. Prostate volume is reduced by 30%. **c**, **f** Month 12: bilateral hypointense scar tissue is present (*arrow*). Prostate volume is reduced by 51%

The MRI control performed approximately 1 h after the TPLA showed in all cases adequate site of treatment. MRI follow-up demonstrated temporal evolution of treatment outcome during the different endpoints: it allows the quantification of the thermal damage extension, the development of the necrosis area with its relative volumetric reduction over time and progressive reduction of the prostate volume (Figs. 2 and 3).

Data analysis shows from pre-treatment to month 12 there was a mean prostate adenoma reduction of 53%. The automatic segmentation software analysis demonstrated a mean volumetric reduction of the necrosis area of 71% from month 1 to month 12 (Fig. 5). In 6 patients with low prostatic volume at pre-treatment (< 40 mL), volume reduction was not relevant in the follow-up with a mean volume reduction of ≤ 8% but with clinical and functional improvement in all cases.

All patients, after 12-month of follow-up, reported a decrease in LUTS with a mean IPSS of 6.2 ± 3.8 and a mean I-QoL of 2.1±1.1. Uroflowmetry showed a mean *Q*_max_ of 16.2 ± 4.9 mL/s and a PVR of 18.8 ± 8.5 mL (all mean ± standard deviation). None of the patients complained of worsening erectile function. That increased in 12 patients at the 3-month clinical assessment and was preserved in the entire population of patients, without significant improvement and with a mean IIEF-5 rate at month 12 of 22.0 ± 3.2 (+ 4%).

Ejaculatory function, evaluated using the MSHQ-EjD, did not worsen in any of the patients through month 12 (MSHQ-EjD 7.7 ± 3.2, mean ± standard deviation), showing improvement in mean score (*p* = 0.030).

TPLA provided adequate improvement of symptoms during the 1-year follow-up in all treated patients and additional invasive treatments were not necessary.

Adverse events, evaluated using the Clavien-Dindo classification, were as follows: 1 case of prolonged haematuria, 7 days after the treatment (grade I); 1 case of orchitis, medically resolved (grade II); and 1 case of bilateral prostatic abscess, successfully treated with percutaneous drainage and 30-day cycle of antibiotic treatment (grade III). This demonstrates an overall adverse event rate of 3/44 (6.8%), without permanent sequelae.

We calculated the frequencies of the main radiological findings for every follow-up step and the results are reported in Table 4.

**Table 4:** Magnetic resonance imaging findings at 1, 3, 6, and 12 months after the procedure (patients, *n* = 44)

Time/findings	Visualisation of fibre track	Post-treatment cavity	Urethra preservation	Periurethral oedema	Prostate volume reduction
1 month	43 (97%)	41 (93%)	44 (100%)	42 (95%)	2 (5%)
3 months	37 (84%)	36 (81%)	44 (100%)	31 (100%)	12 (27%)
6 months	29 (65%)	30 (68%)	44 (100%)	11 (70%)	21(48%)
12 months	22 (50%)	13 (30%)	44 (100%)	3 (7%)	38 (89%)

The main findings observed at 1 month were the laser fibre tracks surrounded by hypointense elliptical-shaped charred tissue, a preserved morphology of the urethra associated with periurethral oedema. Also evident are rare signs of coagulative necrosis at the rim of a cavity filled with blood derivates and proteinaceous fluid. At this time of follow-up, the prostate volume not reduced (volume reduction measured by automated segmentation software mean value 0% in exception of two cases).

At 3 months, we observed bilateral and symmetrical ellipsoid cavitation filled with proteinaceous fluid and blood derivatives around the urethra with signs of necrosis. Even in this follow-up step, the urethra morphology is preserved but it is surrounded by oedema. It is also appreciated a T2-weighted hypointense interface rim along the needle tracks and an initial reduction of prostate volume (volume reduction measured by automated segmentation software mean value 15%).

At 6 months, we observed the reabsorption of the fluid-filled cavities, with a preserved urethral morphology and the periurethral oedema is still evident as like as the T2-weighted hypointense interface rim along the needle tracks. Prostate volume starts to decrease by about 30%.

At the last follow-up (12 months), we observed a T2W hypointense scar tissue that replaced the bilateral cavity with slightly visible signs of fibre tracks. The prostatic urethra is well depicted along with the bladder neck but periurethral oedema persists. At this time, the prostate volume is reduced by at least 40% (volume reduction measured by automated segmentation software; mean value 53%).

The presence of needle tracks was a statistically significant characteristic from month 1 to month 12 (*p* < 0.026) as the presence of periurethral oedema (*p* < 0.002). The observation of a post-treatment cavity becomes statistically significant from month 3 to month 12 (*p* < 0.030) as like prostate volume reduction (*p* < 0.031).

## Discussion

In patients suffering from BPO, long-term medical management incurs adverse events along with a low compliance rate. If pharmacotherapy is insufficient and/or not well tolerated, minimally invasive approaches can be performed. TURP is still considered the cornerstone of BPO minimally invasive treatment [1–4] although it is associated with complications including long recovery time, transient worsening of pre-procedural symptoms and urinary retention due to prostatic oedema [20, 21]. TURP causes a significant incidence of retrograde ejaculation as well [22]. In patients who are still sexually active, the loss of ejaculatory function can be relevant and is associated with considerable discomfort and a lower quality of life [23].

New ablation modalities that may mitigate these side effects include transurethral vaporisation of the prostate, laser enucleation (HoLEP or ThuLEP), photoselective vaporisation of the prostate, transurethral microwave therapy, water vapour thermal therapy, aquablation and prostatic urethral lift. However, HoLEP causes retrograde ejaculation in 70% of cases, whereas the incidence of this adverse effect in the case of photoselective vaporisation has been reported to be 63%, which are rates of retro-ejaculation not much different from that observed with TURP. Therefore, these procedures have appeared as alternatives to TURP to reduce invasiveness and hospitalisation time. Conversely, TPLA demonstrated a 0% rate of retrograde ejaculation proving to be a useful alternative treatment in young men [24–27]. Recently introduced prostatic artery embolisation demonstrated satisfactory results in terms of retrograde ejaculation rate [28–30]. However, its results regarding prostate volume, IPSS and QoL appear to be similar to those provided by other mini-invasive techniques including TPLA.

In comparison, TPLA has some crucial technical and clinical benefits. There is no radiation exposure due to the US or MRI guidance, which also makes it a more approachable technique not requiring a vascular transfemoral route and no need for angiographic equipment. Moreover, it is a short outpatient procedure not limited by anatomical vascular variation and does not cause rectal injuries.

Data collected from our study demonstrated that the main procedural outcomes in terms of PSA and volume reduction as well as symptom relief (*Q*_max_, PVR, and IPSS improvement) are aligned with those obtained with surgical approaches and improved compared to those reported with enucleation techniques. In terms of safety (sexual function sparing, no retrograde ejaculation, low adverse events and complication rate), TPLA appears to be superior to surgical invasive techniques [27]. Patients not only had improvement in urinary symptoms but also had preserved sexual and erectile function as well as improved ejaculatory function at the 12-month follow-up. Previously collected data on 160 patients treated with TPLA for BPH with 1-year follow-up showed a significant improvement of IPSS, PVR, *Q*_max_, QoL, and volume with this technique, with results comparable to those obtained in this study [31–33]. Of note, we evaluated the entire prostate and cavum of ablation volume using MRI follow-up, obtaining a more objective and accurate evaluation of this parameter by monitoring the morphological changes of the treated prostate over time. DWI data are not reported in the current report. These achievements were associated with effective treatment of the underlying pathology allow consideration of TPLA as an option as a first-line therapeutic approach for patients with LUTS caused by BPH. TPLA allows for short recovery time, without general anaesthesia requirement or sedation and provides a safe alternative in patients with coagulation disorders.

**Fig. 4:**
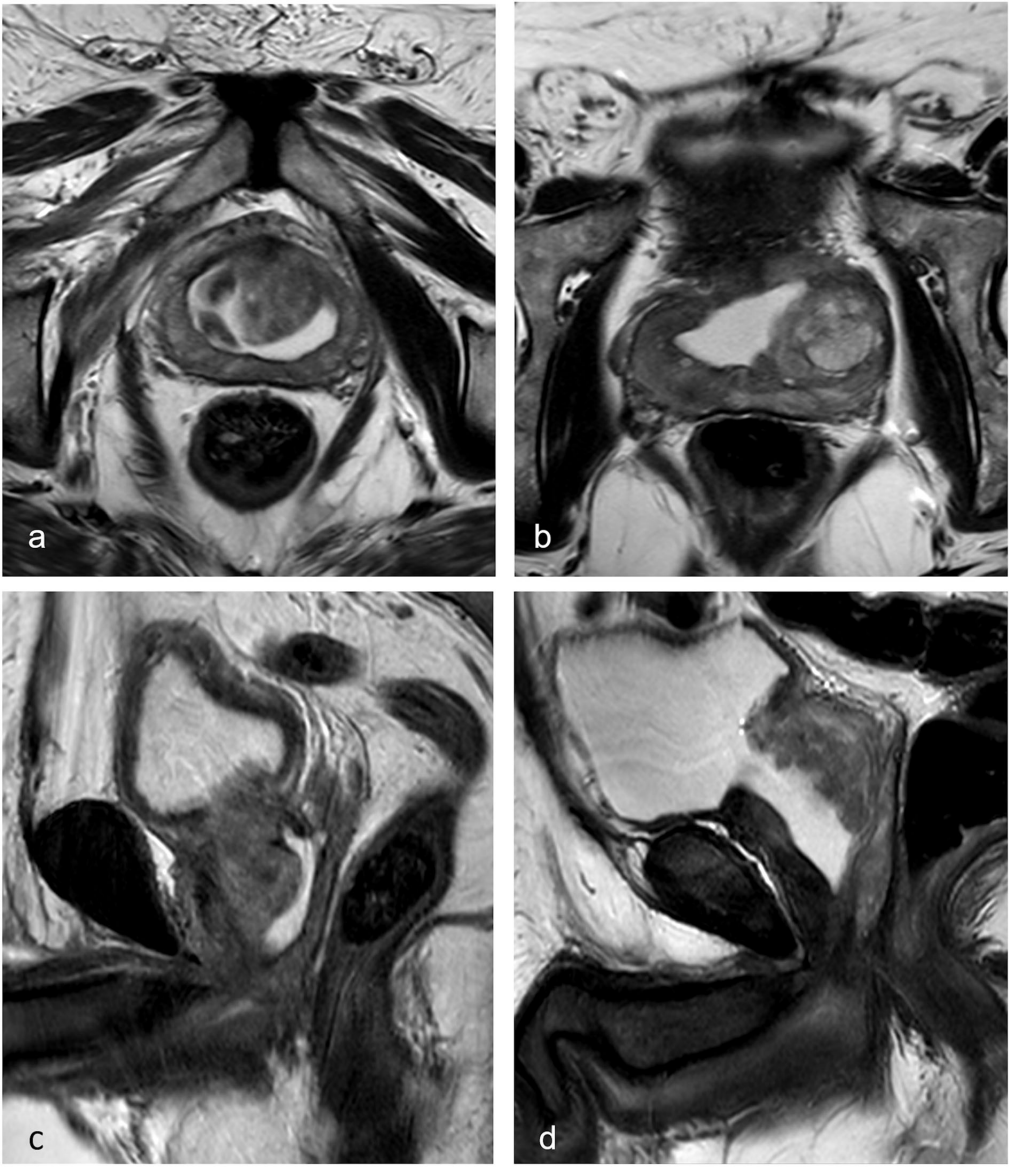
T2-weighted sequences along the axial (**a**) and sagittal (**c**) plane at month 12 after transperineal prostate laser ablation (TPLA) in a 70-year-old patient in comparison with the same sequences (**b**, **d**) in a 68-year-old patient 12 months after transurethral resection of the prostate (TURP). After TPLA, a horseshoe-shaped hyperintense fluid collection in the transitional gland is well emarginated by the prostate pseudocapsule. The urethra is visible as a hyperintense spot inside the solid stromal adenoma core with reduced compression from the hypertrophic central gland. Both bladder neck and urethra morphology are preserved and therefore bladder function and ejaculation are preserved as well. After transurethral resection of the prostate (TURP), the bladder neck is wedged into the surgical cavum. The urethra is no longer visible. Bladder function and ejaculation are impaired

Anatomically, TPLA preserves the prostatic urethra and avoids the bladder neck wedging into the resected ablation area which is seen at MRI in post-TURP (or enucleation treatments) (Fig. 4). TPLA also preserves bladder function and improved urodynamic scores. An additional advantage is that, even if a patient requires additional treatment, standard options can still be implemented after TPLA treatment. TPLA is a low-cost treatment and can be proposed in an outpatient setting at least in selected patients. In our study, it was performed transperineally under US guidance, but a transrectal access with MRI guidance is also available and reported in literature, although less accessible and associated with longer treatment times and higher costs [34].

**Fig. 5:**
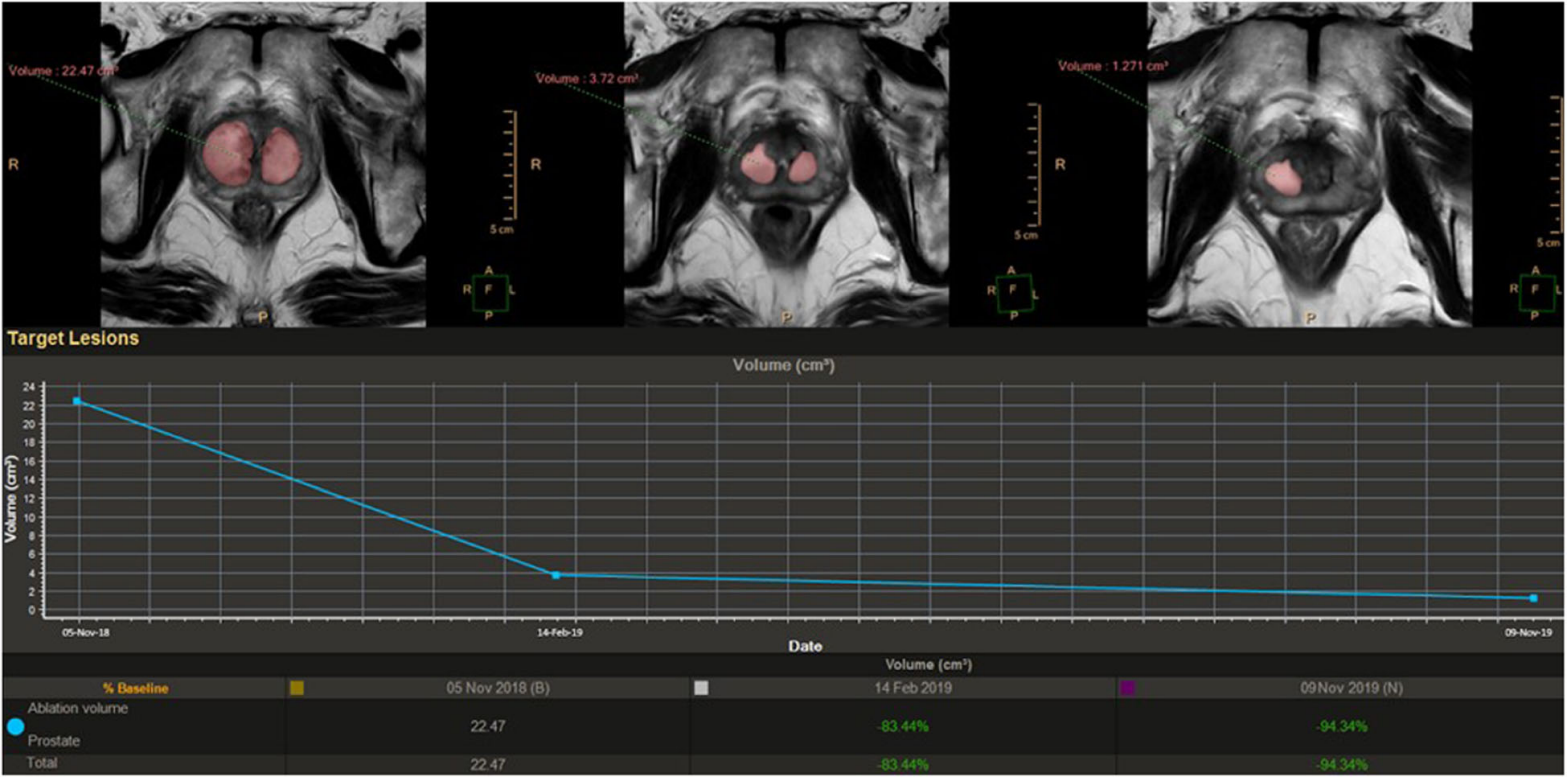
A representative case of automatic segmentation software analysis (Philips IntelliSpace Portal 7.0 Multi Modality Tumor Tracking) of fluid-filled postablation cavities and prostate volumetric trend on axial T2-weighted sequences in a 58-year-old patient between (from left to right) month 1, month 3, and month 12. The right necrotic cavity showed a volumetric reduction of 83.8% between month 1 and month 3 and an 88.8% reduction between month 3 and month 12. The volume of the left cavity showed a reduction of 80.8% between month 1 and month 3, becoming no longer detectable at month 12

Immediate post-treatment effects were demonstrated using MRI, usually evident only as linear hyperintensity surrounded by hypointense elliptical-shaped charred tissue at the site of laser fibre tracks (Fig. 2b). At month 3, large bilateral cavitations filled with haemorrhage and proteinaceous fluid blood derivatives are arranged around the urethra but separated, with ellipsoid morphology on the longitudinal axis (Fig. 5). A hypointense rim as interfaced charred tissue is shown at the edge of haemorrhagic necrosis (Fig. 3a, d). These collections account for an eventual inconsistent volume reduction at this time although tissue is reduced because dense stromal nodular tissue is converted into low-density fluid, with a consequent reduced compression over the urethra and bladder neck and a substantial realignment of the urethral axis at 12 months. From month 3, the volumetric reduction of the prostate becomes quantifiable as progressive up to month 12. In fact, at month 3 and month 6, filled cavities not communicating with the urethra are reabsorbed (Fig. 3b, e). The hypointense interface rim is now visible along the needle tracks. At month 12, tracks from the fibre applicators are slightly visible. Bilateral hypointense scar tissue is present (Fig. 3c, f). Of note, previous studies are limited by the use of US as the dominant follow-up imaging method leading to lower accuracy in the imaging validation of the procedure when compared to our MRI approach.

Regarding the operators’ experience with TPLA, it was approximately 3 years in our study, but through experience, there has been a reduction in procedural time and patient’s hospital stay. In fact, as is for most novel interventional techniques, TPLA requires trained operators. The lack of its diffusion so far limits its usage. Other general limitations of TPLA include the exclusion of patients with large median lobes and those with previous or current urological malignancies. Limitations of this study include the restricted number of patients and the lack of longer-term follow-up. Nevertheless, further investigation with a long-term prospective multicentric study is ongoing. Another limitation is the lack of comparison with standard therapies, beyond the aim of this study.

In conclusion, at 12-month clinical and MRI evaluation, TPLA has demonstrated to be a safe, manageable and effective treatment for BPH in selected patients who cannot tolerate medical treatment and are unfit for other mini-invasive therapies or surgery. The treatment is well tolerated with low morbidity scores, feasible and easy to perform in an outpatient setting with local anaesthesia. Patients are discharged within a few hours on the same day, with an effective improvement of LUTS. The few post-operatory and delayed adverse events were all managed and solved with no sequelae. Based on this and previous experiences, if performed by trained operators, US-guided TPLA should be considered a robust alternative treatment for LUTS in patients with BPH, also considering the COVID-19 pandemic scenario.

## Data Availability

The authors confirm that the data supporting the findings of this study are available within the article.

## Abbreviations

BPH: Benign prostate hyperplasia
BPO: Benign prostatic obstruction
HoLEP: holmium laser enucleation of the prostate
IIEF5: International index of erectile function 5
IPSS: International prostate symptom score
I-QoL: International Quality of life
LUTS: lower urinary tract symptoms
MRI: Magnetic resonance imaging
MSHQ-EjD: Male sexual health questionnaire - ejaculatory dysfunction
PSA: Prostatic specific antigen
PVR: Post-voiding residual
TPLA: Transperineal laser ablation
TURP: Transurethral resection of the prostate
US: Ultrasound
